# Familial hypercholesterolemia care by Dutch pediatricians—mind the gaps

**DOI:** 10.1007/s00431-024-05645-w

**Published:** 2024-06-18

**Authors:** Sibbeliene E. van den Bosch, Barbara A. Hutten, Shirin Ibrahim, Albert Wiegman, Jing Pang, Gerald F. Watts, Willemijn E. Corpeleijn

**Affiliations:** 1 Department of Pediatrics, Amsterdam Cardiovascular Sciences, Amsterdam Gastroenterology Endocrinology Metabolism, Amsterdam University Medical Center, Location University of Amsterdam, Meibergdreef 9, 1105 AZ Amsterdam, The Netherlands; 2 Department of Epidemiology and Data Science, Amsterdam University Medical Center, Location University of Amsterdam, Meibergdreef 9, 1105 AZ Amsterdam, The Netherlands; 3 Department of Vascular Medicine, Amsterdam University Medical Center, Location University of Amsterdam, Meibergdreef 9, 1105 AZ Amsterdam, The Netherlands; 4 Amsterdam Cardiovascular Sciences Research Institute, Diabetes & Metabolism, Amsterdam, The Netherlands; 5 Medical School, University of Western Australia, Perth, WA Australia; 6 Department of Cardiology, Royal Perth Hospital, Perth, WA Australia

**Keywords:** Familial hypercholesterolemia, Children, Pediatricians, Lipid-lowering treatment, Gaps

## Abstract

**Purpose:**

Familial hypercholesterolemia (FH) leads to elevated low-density lipoprotein cholesterol levels, which increases the risk of premature atherosclerotic cardiovascular disease (ASCVD). Since the first functional and morphologic changes of the arterial wall occur in childhood, treatment should start early in childhood to mitigate the elevated risk of ASCVD. Pediatricians play an important role in the detection and care of children with FH. In this study, we aim to explore potential gaps in FH care amongst Dutch pediatricians, in order to enhance their knowledge and awareness of detecting and treating children with FH.

**Methods:**

An anonymous online survey, deployed using Google Forms, including 26 closed and semi-closed questions on FH care in children was distributed by the Dutch Association of Pediatrics via a newsletter to which the majority of the practicing Dutch pediatricians subscribe. In addition, we requested that the pediatric departments of all Dutch hospitals in the Netherlands distribute this survey personally among their employed pediatricians. Respondents were instructed to answer the questions without any help or use of online resources.

**Results:**

Between September 1st, 2023 and November 1st, 2023, 158 (an estimated 11% response rate) Dutch pediatricians completed the survey. They reported a median (IQR) of 15.0 (6.0–22.0) years of experience as a pediatrician, and 34 (21.5%) were working in academic hospitals. The majority (76.6%) of pediatricians correctly identified a typical FH lipid profile but 68 (43.0%) underestimated the true prevalence of FH (1:300). Underestimation and unawareness of the increased risk of FH patients for ASCVD were reported by 37.3% and 25.9% of pediatricians, respectively. Although 70.9% of the pediatricians correctly defined FH, only 67 (42.4%) selected statins and ezetimibe to treat severe hypercholesterolemia.

*Conclusions:*The results of this study suggest significant gaps in knowledge and awareness of FH in children among Dutch pediatricians. FH care in children needs improvement through educational and training initiatives to mitigate the life-long risk of ASCVD from early life.

**Table:** 

**What is Known:**
*• Familial hypercholesterolemia (FH) leads to elevated LDL-cholesterol levels, which increases the risk of premature atherosclerotic cardiovascular disease (ASCVD).* *• The process of atherosclerosis starts in childhood* *• Pediatricians play an important role in the detection and treatment of children with FH.*
**What is New:**
*• Our results highlight significant gaps in care for children with FH amongst pediatricians and this may lead to suboptimal detection and treatment.* *• FH care in children needs improvement by educational initiatives to ultimately prevent ASCVD in adulthood.*

**Supplementary Information:**

The online version contains supplementary material available at 10.1007/s00431-024-05645-w.

## Introduction

Heterozygous familial hypercholesterolemia (FH) is the most common monogenic disorder of cholesterol metabolism and leads to elevated low-density lipoprotein cholesterol (LDL-C) levels from birth. As a result, patients with FH have a 22-fold increased risk of atherosclerotic cardiovascular disease (ASCVD) compared with individuals with normal LDL-C levels without FH [1]. This risk can be mitigated by an observant pediatrician who requests a plasma lipid profile in a child or adolescent with a family history of premature cardiovascular disease (CVD) to diagnose FH.

The first functional and morphologic changes of the arterial wall in FH occur in childhood [2]. Initiation of lipid-lowering treatment (LLT) should start in childhood to reduce these arterial changes and reduce the risk of CVD in adulthood [3]. Therefore, LLT from the age of 8 years old is recommended if lifestyle interventions (exercise, healthy diet, healthy BMI) are insufficient to reach the age-specific LDL-C target goals (8–10 years, LDL-C < 4.0 mmol/L; > 10 years LDL–C < 3.4 mmol/L) [4, 5]. The first pharmacological step is oral LLT: statins with or without ezetimibe. Statins (e.g., rosuvastatin, simvastatin, pravastatin) and ezetimibe are both widely available, effective, safe, and inexpensive. With insufficient LDL-C reduction and/or side effects of conventional therapy, there are novel treatment options, including monoclonal antibodies against proprotein convertase subtilisin/kexin type 9 (PCSK9) [5].

According to a study involving 42,000 adults in 56 countries, patients with FH are frequently diagnosed far too late (median age [interquartile range, IQR] 44.4 [32.5–56.5] years); and only 2.1% are diagnosed in childhood [6, 7]. Based on the worldwide prevalence of FH (1:311 individuals worldwide) around 500,000 children with FH live in Europe of whom 95% remain unidentified [8, 9]. Underdiagnosis leads to suboptimal care of patients who are at high risk of ASCVD. In some countries, including the Netherlands, thousands of patients with FH have been detected through successful national screening programs [10].

The International Atherosclerosis Society (IAS) recommends that diagnosis of FH in children should ideally be made by a pediatrician with training and expertise in lipidology [11]. Treatment of children with FH is ideally a collaboration between general pediatricians, who can treat well-controlled and lower-complexity FH patients, and specialized lipid clinics where more complex FH patients can be cared for [11]. Recently, a survey among Dutch general practitioners revealed significant gaps in knowledge, awareness, and practices that require improvement to enhance the detection and treatment of FH care in adults [12, 13]. No studies have been conducted to explore whether such gaps also extend to pediatricians. Therefore, this study aimed to investigate potential gaps in knowledge, awareness, and practices related to FH care in children and adolescents among Dutch pediatricians.

## Methods

### Study design and participants

On September 1st, 2023 an anonymous online survey was distributed to Dutch pediatricians in the Netherlands via the newsletter of the Dutch Association of Pediatrics *(in Dutch: Nederlandse Vereniging voor Kindergeneeskunde*) to which the majority of the approximately 1500 practicing pediatricians in the Netherlands subscribe. In addition, we requested pediatric departments of all 106 (98 general and 8 academic) hospitals in the Netherlands to distribute this survey to the pediatricians working in those hospitals. The explicit instruction for the survey was to answer the questions without any help. Respondents who indicated that they had not completed their pediatric training were excluded from the study. As questioning healthcare professionals falls outside the scope of the medical ethical committee, local approval was not required.

### Survey details

We adapted a questionnaire previously used in the “ten countries study” [13] in which physicians’ knowledge, awareness, practices, and preferences regarding the care of FH in the Asia–Pacific region were determined. As this formal questionnaire was mainly focused on adults with FH, minor adjustments were made to make the questions applicable to pediatricians and the care of FH in children. The translation of the original English FH questionnaire into Dutch was reviewed by an independent researcher by reverse translation. Demographic questions were added to gain insight into the background of the respondents. The questionnaire, deployed using Google Forms, consisted of 26 closed and semi-closed questions including demographic questions (*n* = 7) and questions specific for FH in children relating to awareness (*n* = 3), knowledge (*n* = 8), practices (*n* = 6), and preferences (*n* = 2) (English and Dutch version of the questionnaire are provided in Supplementary information)*.* Knowledge questions were scored with 1 point per correct answer (maximum score 8 points). If a question was answered with ‘unknown’ or partially answered correctly, the response was considered “incorrect”. Unclear answers on semi-closed questions (for example ‘I have many years’ experience as a pediatricians”) were labeled as ‘no answer’. If a range of numbers was provided (for example ‘I have 20–25 years of experience as pediatrician), the average (i.e., 23 years) was used in the analysis.

### Statistical analyses

Categorical data were presented as proportions (%), while continuous variables were expressed as means (standard deviation [SD]) for normally distributed variables and medians (interquartile rate [IQR]) for skewed distributions. Differences in knowledge scores between groups (sex, hospital category, and those with FH children in their practice) were analyzed using the independent sample *t* test, and the association between knowledge scores and years of experience as a pediatrician was analyzed using linear regression. Two-sided *p* values < 0.05 were considered statistically significant. All analyses were performed using IBM SPSS version 28 (SPSS Inc., Chicago, IL, USA).

## Results

### Description of the study population

Between September 1st and November 1st, 2023, a total of 158 respondents (an estimated 11% response rate) that had completed their pediatric training (71.5% female) successfully completed the online questionnaire. The demographic characteristics of the pediatricians are summarized in Table 1. They reported a median (IQR) experience of 15.0 (6.0–22.0) years in pediatrics. All twelve provinces in the Netherlands were represented by the respondents with 34 (21.5%) of pediatricians working in academic hospitals and 124 (78.5%) pediatricians in general hospitals.

**Table 1:** Characteristics of the study population

	Respondents *n* = 158
Completed pediatrician training—no. (%)	158 (100)
Female sex—*n* (%)	113 (71.5)
Experience as pediatrician in years—median (IQR)	15.0 (6.0–22.0)
Children load per month—median (IQR)	90.0 (51.3–125.0)
Children with FH in practice—yes (%)	90 (57)
*Practice setting*—*n* (%)	
Academic hospital General hospital	34 (21.5) 124 (78.5)
*Hospital location*—*n* (%)	
Drenthe Flevoland Friesland Gelderland Groningen Limburg Noord-Brabant Noord-Holland Overijssel Utrecht Zeeland Zuid-Holland	5 (3.2) 5 (3.2) 5 (3.2) 16 (10.1) 11 (7.0) 10 (6.3) 26 (16.5) 36 (22.8) 3 (1.9) 14 (8.9) 3 (1.9) 24 (15.2)

*FH* familial hypercholesterolemia, *n* number, *IQR* interquartile range

### FH awareness

As shown in Table 2, 111 pediatricians (70.3%) rated themselves with an above-average knowledge score (≥ 4 out of 7). While only 83 (52.5%) of the respondents indicated awareness of the current FH guidelines, the majority (*n* = 127, 80.4%) of pediatricians knew to which specialized lipid disorder health clinic they could refer patients.

**Table 2:** Summary of questionnaire findings amongst Dutch pediatricians on FH care in children

Question	Pediatricians *n* = 158
Awareness	
FH familiarity rated ≥ 4—*n* (%) Aware of FH guidelines—*n* (%) Aware of lipid disorder health institutes—*n* (%)	111 (70.3) 83 (52.5) 127 (80.4)
Knowledge	
Correctly defined FH—*n* (%) Correctly identified FH lipid profile—*n* (%) Correctly identified FH prevalence—*n* (%) Correctly identified FH transmission rate to first-degree relatives—*n* (%) Correctly identified that genetic testing is not required for an FH diagnosis—*n* (%) Correctly identified CVD risk in untreated FH patients—*n* (%) Correctly identified age of premature CVD in males—*n* (%) Correctly identified age of premature CVD in females—*n* (%)	112 (70.9) 121 (76.6) 44 (27.8) 101 (63.9) 72 (45.6) 42 (26.6) 9 (5.7) 6 (3.8)
Practice	
Assesses family history of CAD in children with a myocardial or cerebral infarction—*n* (%) Screens lipid profile in close relatives of children with FH—*n* (%) Performs screening for hypercholesterolemia in children in families with premature CAD, age group:	158 (100.0) 101 (63.9)
0–6 years—*n* (%)	25 (15.8)
7–12 years—*n* (%)	106 (67.1)
13–18 years—*n* (%)	15 (9.5)
Unknown—*n* (%)	12 (7.6)
Has referred children to health institutes for lipid disorders (when aware of them)—*n* (%)	83 (65.4)
Prescribes statin monotherapy in case of hypercholesterolemia—*n* (%)	97 (61.4)
Prescribes statin and ezetimibe in case of severe hypercholesterolemia—*n* (%)	67 (42.4)
Preference	
Consider pediatricians for early screening and detection of FH in children—*n* (%) Prefers an alarming comment to lipid profiles at risk for FH—*n* (%)	60 (38.0) 130 (82.3)

*CVD* cardiovascular disease, *CAD* coronary artery disease, *FH* familial hypercholesterolemia, *no.* number, *IQR* interquartile range

### FH knowledge

None of the respondents answered all eight knowledge questions correctly. Pediatricians who rated their knowledge as above average (≥ 4 points out of 7) and those having FH children in their practice scored significantly higher in knowledge scores (mean score [SD] 3.49 [1.60] vs. 2.55 [1.36], *p* < 0.001; and 3.53 [1.53] vs. 2.70 [1.53], *p* = 0.001, respectively). We observed higher knowledge scores among more experienced pediatricians (β 0.032, *p* = 0.021). There were no significant associations between gender (male vs. female) of the pediatricians or practice setting (academic vs. general hospital) with knowledge scores (mean score [SD] 3.31 [1.54] vs 3.17 [1.61], *p* = 0.610; and 3.41 [1.67] vs 3.15 [1.56], *p* = 0.401, respectively).

The majority of pediatricians correctly defined the diagnosis of FH (*n* = 112, 70.9%) and identified the correct plasma lipid profile for FH (121, 76.6%). However, only a quarter of pediatricians (*n* = 44, 27.8%) correctly identified the actual prevalence of FH (1:300). Furthermore, 72 pediatricians (45.6%) knew that FH does not necessarily need to be confirmed through genetic diagnosis. Only a few pediatricians correctly identified the age threshold for premature CVD in men (< 55 years) and women (< 60 years), with 9 (5.7%) and 6 (3.8%) respondents, respectively.

### FH practice, preference, and care

All pediatricians reported that they inquired about a family history of CVD when a child experienced a myocardial infarction or cerebral infarction (Table 2). A majority (*n* = 106, 67.1%) selected to screen children for FH aged 7–12 years, and 9.5% opted for screening at a later age (13–18 years). For children with hypercholesterolemia, 97 (61.4%) pediatricians would prescribe statin monotherapy, and in case of severe hypercholesterolemia, only 67 (42.4%) of the pediatricians would prescribe combination therapy with a statin and ezetimibe. The combination of statins with bile acid sequestrants and/or nicotinic acid, which does not align with current guidelines, was mentioned by 19 (12%) pediatricians. Most pediatricians (130, 82.3%) indicated that an alarming comment on a lipid profile at risk for FH would help optimize the detection of FH. The diagnosis of FH in children should ideally be made by a pediatrician, as also recently stated by the IAS [11]. But only 60 (38.0%) pediatricians considered they were an integral part of the FH detection team, with general practitioners being specified most frequently (*n* = 133, 84.2%).

## Discussion

This study investigated potential gaps in awareness, knowledge, preferences, and practices in the care of children and adolescents with FH amongst Dutch pediatricians, with the ultimate aim of providing Dutch pediatricians with the right knowledge and skills to detect and treat such children. Given the crucial role of pediatricians in detecting and treating children with FH, this study reveals concerning gaps in all the critical domains of FH care in children. To enhance care, comprehensive improvements are necessary to raise FH awareness through the implementation of education and upskilling initiatives.

### Mind the gaps

Ours is the first questionnaire-based study that investigated gaps in FH care for children amongst pediatricians. While there are studies that have surveyed general practitioners on FH, those tend to be more focused on FH care in adults [12–14]. Our results align with previous similar questionnaire-based research involving general practitioners in the Netherlands, although it is noteworthy that Dutch general practitioners perceive themselves as more aware of FH guidelines compared with the pediatricians we surveyed (91.4% vs. 52.5%) [12]. General practitioners also score higher in correctly defining FH (83.7% vs. 70.9%) and in correctly identifying an FH plasma lipid profile (87.8% vs. 76.6%) [12]. Despite FH being the most prevalent monogenic disorder worldwide, many physicians remain unaware of this condition [9, 15]. Pediatricians tend to perform screening in children suspected of having FH at a later age than advised by the European Atherosclerosis Society (EAS), which recommends starting from 5 years old [4, 11]. Fortunately, most pediatricians are aware of the clinical centers of expertise for lipid disorders to where they could refer children with FH. However, considering the importance and effectiveness of treatment for FH, we argue that basic knowledge should be present amongst non-specialized pediatricians to ensure optimal detection and care of affected children.

No knowledge questions received a 100% correct response, and five out of eight knowledge questions were answered correctly by less than half of the pediatricians. This is concerning, especially considering that this questionnaire was performed in the Netherlands, a country that has been a pioneer in FH care and has had a nationwide successful screening program for an extended period [16]. These scores are therefore most likely worse in countries with less emphasis on the detection of FH.

### Clinical implications

Although statins have been proven to be effective and safe for children based on extensive global research, pharmacological therapy of children was under-appreciated by pediatricians. Nonetheless, early treatment of FH is crucial in reducing the risk for ASCVD [3, 4]. To gain a deeper understanding of the causes of existing gaps, e.g. lack of training or accessibility to guidelines, it is essential to conduct qualitative research in the form of interviews and/or focus groups, in order to implement targeted improvements in FH care for children. The responsibility to ensure that pediatricians and pediatric residents have adequate knowledge lies in the hands of the specialized lipid clinic for the specific region.

One of the improvements we propose is to highlight FH in the education program of pediatric residents. Additionally, our center is working on a FH protocol in the local language that should be easily accessible on the Internet, and we organize refresher courses for pediatricians. We expect that these initial steps will close the gaps we have identified.

As mentioned earlier, the results of this study from an international perspective are even more concerning, given that the Netherlands has the highest rate of FH diagnosis in the world [17]. It is likely that knowledge amongst pediatricians is even more suboptimal in other countries where FH is less prioritized. Therefore, we recommend conducting this research in more countries to develop a specific action plan tailored to each country, with the common goal of allowing children with FH to grow up healthy and with the life expectancy of the background population.

### Limitations

Some limitations need to be considered in this study. Although respondents were instructed not to look up answers there is a possibility that pediatricians may have still searched for answers (online), which would have led to an underestimation of the problem. However, this potential underestimation does not change our recommendations to address the existing gaps. Secondly, the question arises whether the respondents are a good representation of pediatricians. Considering that > 10% of the approximately 1500 practicing pediatricians in the Netherlands have completed this questionnaire, with representative sampling in relation to gender, academic/non-academic, and representation from all provinces, we are confident that our sample reasonably represents Dutch pediatricians. In future research, it may be considered to distribute this questionnaire to specialized pediatric nurses and nurse practitioners, as they are also involved in the detection and treatment of FH. Next to FH, questions about other dyslipidemias, such as elevated lipoprotein(a)—another inherited much more common risk factor for ASCVD—can be implemented to explore potential gaps in cardiovascular research management.

## Conclusions

In this research, gaps in awareness, knowledge, and practice of pediatricians regarding FH care were identified in a leading country in the care of FH [17]. These gaps result in suboptimal detection and treatment of children with FH. This certainly is a missed opportunity as most pediatricians will enquire about family history during an initial visit to the outpatient clinic. With adequate knowledge and awareness, this will lead to recognition of a family history suspicious of FH. Further research that focuses on effective strategies to increase awareness and knowledge of FH in primary and secondary healthcare is necessary to improve FH care for children. In the meantime, initial steps in education and the development of local protocols can be established with the aim of ensuring that children and adolescents with FH grow up to be healthy adults.

### Supplementary Information

Below is the link to the electronic supplementary material.

Supplementary information 1: English and Dutch version of the questionnaire. (DOCX 38 KB)

## Data Availability

No datasets were generated or analysed during the current study.

## Abbreviations

ASCVD: Atherosclerotic cardiovascular disease
CVD: Cardiovascular disease
EAS: European Atherosclerosis Society
FH: Familial hypercholesterolemia
LDL-C: Low-density lipoprotein cholesterol
LLT: Lipid-lowering therapy
IQR: Interquartile range
IAS: International Atherosclerosis Society
SD: Standard deviation
