# Supplementary material for: Familial hypercholesterolemia care by Dutch pediatricians—mind the gaps
Source: Eur J Pediatr. 2024 Jun 18;183(9):3877–83. doi: 10.1007/s00431-024-05645-w (PMC11322321; doi:10.1007/s00431-024-05645-w)
Supplement: Supplementary file 1 — Supplementary information 1: English and Dutch version of the questionnaire. (DOCX 38 KB) [file 431_2024_5645_MOESM1_ESM.docx]

**Supplemental appendix**

**Familial hypercholesterolemia care by Dutch pediatricians**

**- Mind the gaps -

by**

Sibbeliene E. van den Bosch, Barbara A. Hutten , Shirin Ibrahim, Albert Wiegman, Jing Pang, Gerald F. Watts, Willemijn E. Corpeleijn

**Table of contents**

**Page**

**FH Pediatrician Survey – English version** 3

**FH Pediatrician Survey – Dutch version** 8

**FH Pediatrician Survey**

**1. Have you completed your training as a pediatrician?**○ Yes
○ No, please do not continue with this questionnaire

**2. On a scale of 1 to 7, how familiar are you with familial hypercholesterolemia, where 1 means 'not familiar' and 7 means 'fully familiar'?**

Not familiar

○ 1

○ 2

○ 3

○ 4

○ 5

○ 6

○ 7

Fully familiar

**3. Are you aware of the current guidelines for the detection and treatment of children with familial hypercholesterolemia?**

○ Yes

○ No

**4. Which of the following statements best describes heterozygous familial hypercholesterolemia?**

○ Having a family member diagnosed with hypercholesterolemia

○ A genetic condition with high cholesterol and a positive family history of early cardiovascular diseases

○ Multiple abnormalities in the lipid profile with a genetic origin

○ A very rare, potentially fatal condition caused by cholesterol levels up to six times the normal value

○ I don't know

**5. Which of the following lipid profiles best fits the diagnosis of familial hypercholesterolemia? (normal values: total cholesterol <5.5 mmol/L; triglycerides <1.7 mmol/L; HDL-cholesterol >1.0 mmol/L; LDL-cholesterol <3.5 mmol/L)**○ Total cholesterol 6.0 mmol/L; triglycerides 3.4 mmol/L; HDL-cholesterol 0.8 mmol/L; LDL-cholesterol 3.8 mmol/L
○ Total cholesterol 6.3 mmol/L; triglycerides 12.2 mmol/L; HDL-cholesterol 1.0 mmol/L; LDL-cholesterol - mmol/L
○ Total cholesterol 8.0 mmol/L; triglycerides 1.1 mmol/L; HDL-cholesterol 1.0 mmol/L; LDL-cholesterol 6.5 mmol/L
○ Total cholesterol 5.4 mmol/L; triglycerides 1.3 mmol/L; HDL-cholesterol 1.7 mmol/L; LDL-cholesterol 3.1 mmol/L
○ Total cholesterol 7.1 mmol/L; triglycerides 1.0 mmol/L; HDL-cholesterol 3.5 mmol/L; LDL-cholesterol 3.2 mmol/L

**6. Which of the following options do you think could further optimize the detection of patients with familial hypercholesterolemia?**

○ Marking an abnormal lipid profile indicative of familial hypercholesterolemia

○ An alert through your local electronic patient system

○ Being informed by the laboratory about patients with an abnormal lipid profile over the phone

○ All of the above

○ None of the above

○ I don't know

○ Other...

**7. What is the prevalence (worldwide) of familial hypercholesterolemia?**
○ 1 in 300
○ 1 in 1000
○ 1 in 3000
○ 1 in 30,000
○ 1 in 300,000
○ I don’t know

**8. What is the likelihood that a first-degree family member (parents, siblings) of a child with familial hypercholesterolemia also has familial hypercholesterolemia?**

○ 0%

○ 25%

○ 50%

○ 75%

○ 100%

○ I don’t know

**9. How much greater is the risk of premature cardiovascular diseases in untreated patients* with familial hypercholesterolemia compared to individuals without familial hypercholesterolemia? *In adults**

○ Twice as high

○ Five times as high

○ Ten times as high

○ 20 times as high

○ 50 times as high

○ I don't know

**10. Up to what age do you consider cardiovascular diseases as 'premature' in MEN? Leave the field blank if you choose 'I don't know.'**

Your answer

**11. Up to what age do you consider cardiovascular diseases as 'premature' in WOMEN? Leave the field blank if you choose 'I don't know.'**

Your answer

**12. What would you do in the case of a child with a myocardial or cerebral infarction? (Select all that apply)**

○ Check for the presence of an arcus cornealis
○ Check for the presence of tendon xanthomas
○ Conduct a detailed family history for cardiovascular diseases
○ Screen immediate family members for hypercholesterolemia
○ All of the above
○ None of the above

**13. Is the following statement true or false?**

Statement:

'Accurately diagnosing familial hypercholesterolemia can only be done through a genetic test'

○ This is true

○ This is false

○ I don't know

**14 How many of your current patients have been diagnosed with familial hypercholesterolemia?**

Your answer

**15. If you encounter a child with familial hypercholesterolemia, who would you screen by determining a lipid profile?**○ The family members of the child
○ The family members and other relatives
○ No one
○ Not applicable

**16. Which healthcare professionals do you think would be most suitable for early detection of patients with familial hypercholesterolemia and screening first-degree family members? (Choose up to two from the options below)**

○ Vascular internist

○ General practitioner (GP)

○ Cardiologist

○ Nurse experienced in cardiovascular disease prevention

○ Pediatrician

○ Gynecologist/obstetrician

○ Endocrinologist

○ Other:...

**17. At what age would you test children for hypercholesterolemia in families with premature cardiovascular diseases?**

○ 0-6 years

○ 7-12 years

○ 13-18 years

○ None of the above

○ I don’t know

**18. Are you familiar with specialized healthcare institutions to which you could refer children with lipid disorders?**

○ No

○ Yes*

***19.** *If yes in 18* **Have you ever referred children with familial hypercholesterolemia to such a healthcare institution?**

○ Yes

○ No

**20. What medication would you prescribe for children to treat hypercholesterolemia? (Select all that apply)**

○ Bile acid sequestrant

○ Ezetimibe

○ Statins

○ Fibrates

○ Nicotinic acid

○ None of the above

○ Other:...

**21. Which combination of medications would you prescribe to treat severe hypercholesterolemia in children? (Select all that apply)**

○ Statin + bile acid sequestrant

○ Statin + nicotinic acid

○ Statin + ezetimibe

○ Statin + ezetimibe + nicotinic acid

○ Statin + ezetimibe + bile acid sequestrant

○ None of the above

○ Other:...

**22. What is your gender?**

○ Female

○ Male

○ Prefer not to say

**23. In which province is your hospital located?**○ Drenthe
○ Flevoland
○ Friesland
○ Gelderland
○ Groningen
○ Limburg
○ Noord-Brabant
○ Noord-Holland
○ Overijssel
○ Utrecht
○ Zeeland
○ Zuid-Holland

**24. Do you work in a general of an academic hospital?**○ Academic
○ Peripheral
○ Other:...

**25. How many years of experience do you have as a pediatrician?**

Your answer

**26. How many patients do you see approximately per month (for all conditions)?**
Your answer

**Vragenlijst voor kinderartsen over FH**

**1. Heeft u uw opleiding tot kinderarts voltooid?**○ Ja
○ Nee, gaarne deze vragenlijst niet verder invullen

**2. Op een schaal van 1 t/m 7, hoe bekend bent u met familiaire hypercholesterolemie, waarbij 1 staat voor 'niet bekend' en 7 staat voor 'optimaal bekend'?**

Niet bekend

○ 1

○ 2

○ 3

○ 4

○ 5

○ 6

○ 7

Optimaal bekend

**3. Bent u op de hoogte van de huidige richtlijnen voor opsporing en behandeling van kinderen met familiaire hypercholesterolemie?**

○ Ja

○ Nee

4. **Welk van onderstaande stellingen beschrijft heterozygote familiaire hypercholesterolemie het beste?**

○ Het hebben van een familielid waarbij hypercholesterolemie is vastgesteld

○ Een genetische aandoening met een hoog cholesterol en een positieve familieanamnese voor vroege hart- en vaatziekten

○ Meerdere afwijkingen in het lipidenprofiel die een genetische oorsprong kunnen hebben

○ Een zeer zeldzame, potentieel fatale aandoening, veroorzaakt door cholesterolwaarden die tot wel zes keer de normaalwaarde kunnen bereiken

○ Weet ik niet

**5. Welk van onderstaande lipidenprofielen past het beste bij de diagnose familiaire hypercholesterolemie? (normaalwaarden: totaal cholesterol <5.5 mmol/L; triglyceriden <1.7 mmol/L; HDL-cholesterol >1.0 mmol/L; LDL-cholesterol <3.5 mmol/L)**
○ Totaal cholesterol 6.0 mmol/L; triglyceriden 3.4 mmol/L; HDL-cholesterol 0.8 mmol/L; LDL-cholesterol 3.8 mmol/L
○ Totaal cholesterol 6.3 mmol/L; triglyceriden 12.2 mmol/L; HDL-cholesterol 1.0 mmol/L; LDL-cholesterol - mmol/L
○ Totaal cholesterol 8.0 mmol/L; triglyceriden 1.1 mmol/L; HDL-cholesterol 1.0 mmol/L; LDL-cholesterol 6.5 mmol/L
○ Totaal cholesterol 5.4 mmol/L; triglyceriden 1.3 mmol/L; HDL-cholesterol 1.7 mmol/L; LDL-cholesterol 3.1 mmol/L
○ Totaal cholesterol 7.1 mmol/L; triglyceriden 1.0 mmol/L; HDL-cholesterol 3.5 mmol/L; LDL-cholesterol 3.2 mmol/L

**6. Welk van de volgende opties denkt u dat de opsporing van patiënten met familiaire hypercholesterolemie verder zou kunnen optimaliseren?**

○ Een markering bij een afwijkend lipidenprofiel alarmerend voor familiaire hypercholesterolemie

○ Een alert via uw eigen klinische software systeem

○ Telefonisch door het laboratorium gewezen worden op patiënten met een afwijkend lipidenprofiel

○ Alle bovenstaande

○ Geen van bovenstaande

○ Weet ik niet

○ Anders…

**7. Wat benadert de prevalentie (wereldwijd) van familiaire hypercholesterolemie het beste?**

○ 1 op de 300

○ 1 op de 1000

○ 1 op de 3000

○ 1 op de 30 000

○ 1 op de 300 000

○ Weet ik niet

**8. Hoe groot is de kans dat een eerstegraads familielid (ouders, broers/zussen) van een kind met familiaire hypercholesterolemie ook familiaire hypercholesterolemie heeft?**

○ 0%

○ 25%

○ 50%

○ 75%

○ 100%

○ Weet ik niet

**9. Hoeveel groter is het risico op premature hart- en vaatziekten in onbehandelde patiënten* met familiaire hypercholesterolemie in vergelijking met personen zonder familiaire hypercholesterolemie? *Bij volwassenen**

○ Twee keer zo groot

○ Vijf keer zo groot

○ Tien keer zo groot

○ 20 keer zo groot

○ 50 keer zo groot

○ Weet ik niet

**10. Tot welke leeftijd beschouwt u hart- en vaatziekten als ‘prematuur’ bij MANNEN? Laat het veld leeg als u ‘weet ik niet’ wenst te antwoorden.**

Uw antwoord

**11. Tot welke leeftijd beschouwt u hart- en vaatziekten als ‘prematuur’ bij VROUWEN? Laat het veld leeg als u ‘weet ik niet’ wenst te antwoorden.**

Uw antwoord

**12. Welk van de volgende opties zou u uitvoeren bij een kind met een myocard- of herseninfarct? (Meerdere opties mogelijk, kruis alles aan wat van toepassing is)**
○ Kijken naar aanwezigheid van een arcus cornealis
○ Kijken naar aanwezigheid van pees xanthomen
○ Een gedetailleerde familieanamnese voor hart- en vaatziekten uitvragen
○ Naaste familieleden screenen voor hypercholesterolemie
○ Alle bovenstaande
○ Geen van bovenstaande

**13. Is de volgende stelling juist of onjuist?**

Stelling:

‘Het accuraat stellen van de diagnose familiaire hypercholesterolemie kan alleen via een genetische test’

○ Dit is juist

○ Dit is onjuist

○ Weet ik niet

**14. Hoeveel van uw huidige patiënten zijn gediagnosticeerd met familiaire hypercholesterolemie?**


Uw antwoord

**15. Als u een kind ziet met familiaire hypercholesterolemie, wie zou u screenen middels het bepalen van een lipidenprofiel?**
○ De gezinsleden van het kind
○ De gezinsleden en overige familieleden
○ Niemand
○ Niet van toepassing

**16. Welke zorgverleners zouden, in uw optiek, het meest geschikt zijn voor het vroeg opsporen van patiënten met familiaire hypercholesterolemie en het screenen van eerstegraads familieleden? (Kies maximaal twee van onderstaande)**

○ Vasculaire internist

○ Huisarts

○ Cardioloog

○ Verpleegkundige die ervaring heeft met preventie van hart- en vaatziekten

○ Kinderarts

○ Gynaecoloog/obstetricus

○ Endocrinoloog

○ Anders:…

**17. Op welke leeftijd zou u kinderen testen op hypercholesterolemie in families met premature hart- en vaatziekten?**

○ 0-6 jaar

○ 7-12 jaar

○ 13-18 jaar

○ Geen van bovenstaande

○ Weet ik niet

**18. Bent u bekend met gespecialiseerde zorginstellingen waar u kinderen met lipiden aandoeningen naar zou kunnen verwijzen?**

○ Nee

○ Ja

**19.** *Indien Ja bij vraag 18* **Heeft u kinderen met familiaire hypercholesterolemie weleens verwezen naar een dergelijke zorginstelling?**

○ Ja

○ Nee

**20. Welk medicament zou u voorschrijven bij kinderen om hypercholesterolemie te behandelen ? (Selecteer alles wat van toepassing is)**

○Galzuurbinder

○Ezetimibe

○Statines

○Fibraten

○Nicotinezuur

○Geen van bovenstaande

○Anders:…

**21. Welke combinatie van medicamenten zou u voorschrijven om ernstige hypercholesterolemie bij kinderen te behandelen? (Selecteer alles wat van toepassing is)**

○Statine + galzuurbinder

○Statine + nicotinezuur

○Statine + ezetimibe

○Statine + ezetimibe + nicotinezuur

○Statine + ezetimibe + galzuurbinder

○Geen van bovenstaande

○ Anders:…

**22. Wat is uw geslacht?**

○ Vrouw

○ Man

○ Vermeld ik niet

**23. In welke provincie bevindt uw ziekenhuis zich?**
○ Drenthe
○ Flevoland
○ Friesland
○ Gelderland
○ Groningen
○ Limburg
○ Noord-Brabant
○ Noord-Holland
○ Overijssel
○ Utrecht
○ Zeeland
○ Zuid-Holland

**24. Is het ziekenhuis waar u werkt perifeer of academisch?**
○ Academisch
○ Perifeer
○ Anders:…

**25. Hoeveel jaar ervaring heeft u als kinderarts?**


Uw antwoord

**26. Hoeveel patiënten ziet u ongeveer per maand (voor alle aandoeningen)?**

Uw antwoord
